# A Kalman Filter for Multilinear Forms and Its Connection with Tensorial Adaptive Filters †

**DOI:** 10.3390/s21103555

**Published:** 2021-05-20

**Authors:** Laura-Maria Dogariu, Constantin Paleologu, Jacob Benesty, Cristian-Lucian Stanciu, Claudia-Cristina Oprea, Silviu Ciochină

**Affiliations:** 1 Department of Telecommunications, University Politehnica of Bucharest, 1-3, Iuliu Maniu Blvd., 061071 Bucharest, Romania; ldogariu@comm.pub.ro (L.-M.D.); cristian@comm.pub.ro (C.-L.S.); cristina@comm.pub.ro (C.-C.O.); silviu@comm.pub.ro (S.C.); 2 INRS-EMT, University of Quebec, Montreal, QC H5A 1K6, Canada; Jacob.Benesty@inrs.ca

**Keywords:** adaptive filters, Kalman filter, multilinear forms, system identification, tensor decomposition

## Abstract

The Kalman filter represents a very popular signal processing tool, with a wide range of applications within many fields. Following a Bayesian framework, the Kalman filter recursively provides an optimal estimate of a set of unknown variables based on a set of noisy observations. Therefore, it fits system identification problems very well. Nevertheless, such scenarios become more challenging (in terms of the convergence and accuracy of the solution) when the parameter space becomes larger. In this context, the identification of linearly separable systems can be efficiently addressed by exploiting tensor-based decomposition techniques. Such multilinear forms can be modeled as rank-1 tensors, while the final solution is obtained by solving and combining low-dimension system identification problems related to the individual components of the tensor. Recently, the identification of multilinear forms was addressed based on the Wiener filter and most well-known adaptive algorithms. In this work, we propose a tensorial Kalman filter tailored to the identification of multilinear forms. Furthermore, we also show the connection between the proposed algorithm and other tensor-based adaptive filters. Simulation results support the theoretical findings and show the appealing performance features of the proposed Kalman filter for multilinear forms.

## 1. Introduction

The identification of multilinear forms (or linearly separable systems) can be efficiently exploited in the framework of different applications, like channel equalization [1,2], nonlinear acoustic echo cancellation [3,4], source separation [5,6], array beamforming [7,8], and object recognition [9,10]. In these contexts, the basic approach relies on tensor decomposition and modeling techniques [11,12,13,14], since the multilinear forms can be modeled as rank-1 tensors. The main idea is to combine (i.e., “tensorize”) the solutions to low-dimension problems, in order to efficiently solve a multidimensional system identification problem, which is usually characterized by a large parameter space. Such scenarios can appear in the framework of multichannel systems, e.g., those with a large number of sensors and actuators, such as in active noise control systems [15], adaptive beamforming [16], microphones arrays [17], etc.

The particular cases of bilinear and trilinear forms have been previously addressed in the literature from a system identification perspective. In [18], an iterative Wiener filter for bilinear forms was developed in the framework of a multiple-input/single-output (MISO) system. Compared to the conventional Wiener solution, the iterative version can obtain a good accuracy of the solution, even when a few data are available for the estimation of the statistics. Furthermore, in [19], this solution was extended to the identification of trilinear forms, based on the decomposition of third-order tensors (of rank-1). Since there are inherent limitations to the Wiener filters (time-invariant framework, matrix inversion operation, etc.), a more appropriate and practical solution relies on adaptive filtering [20,21,22]. Consequently, several adaptive filters tailored to the identification of bilinear and trilinear forms have also been developed, following the main categories of algorithms. For example, the least-mean-square (LMS) and normalized LMS (NLMS) versions can be found in [23,24]. In addition, the recursive least-squares (RLS) algorithms for bilinear and trilinear forms were developed in [25,26], respectively. These algorithms have improved convergence features as compared to their LMS-based counterparts. Moreover, a Kalman filter tailored to the identification of bilinear forms was analyzed in [27,28].

These adaptive solutions are suitable in real-world scenarios, e.g., working in nonstationary environments and/or requiring real-time processing. Among them, the Kalman filter represents a very appealing choice [29,30,31]. As compared to other adaptive filters, where the system to be identified is considered to be deterministic in their derivations, the Kalman filter takes the “uncertainties” in the system into account, and is thus successfully employed in a wide range of applications, e.g., [32,33,34,35,36,37] and the references therein. Recently, in [36], an adaptive Kalman-filter-based variational Bayesian, which achieves a simultaneous estimation of the process noise covariance matrix and of the measurement noise covariance matrix, is presented, with applications in target tracking. In [37], the authors propose a multiple strong tracking adaptive square-root cubature Kalman filter, which can be used to improve the in-flight alignment, with applications in guided weaponry, unmanned automatic vehicles, and robots. The numerous different fields of applicability of the Kalman filter represent the main motivation behind the development presented in this paper, which targets the derivation of such a filter, tailored to the identification of multilinear forms.

Recently, an iterative Wiener filter was designed for multilinear forms [38], followed by the adaptive solutions based on the LMS and RLS algorithms [39,40]. The goal of this paper is twofold. First, it extends the work in [39], by proposing a Kalman filter for multilinear forms, with improved convergence features compared to the LMS-based counterparts. Second, it establishes the connection between the proposed Kalman filter and the tensor-based adaptive algorithms presented in [40], showing how these algorithms can be obtained as simplified versions of the Kalman filter for multilinear forms.

The rest of the paper is organized as follows. In Section 2, we present the system model behind the multilinear framework, which is formulated in the context of an MISO system identification problem. The proposed tensorial Kalman filter for the identification of such multilinear forms is derived in Section 3. Furthermore, in Section 4, we show how this algorithm is connected with the main tensor-based adaptive filters, which belong to the LMS and RLS families. Simulation results provided in Section 5 support the theoretical findings and indicate the good performance of the proposed Kalman filter for multilinear forms. Finally, in Section 6, the main conclusions are outlined, together with several perspectives for future works.

## 2. Multilinear Framework for MISO System Identification

Let us consider a real-valued MISO system with *N* individual channels, which are modeled as finite-impulse-response (FIR) filters of lengths $L_{n},\;n=1,2,\ldots ,N$. The impulse responses of these channels at the discrete-time index *t* are characterized by the vectors $h_{n}\left(t\right)={\left[h_{n,1}\left(t\right)\;h_{n,2}\left(t\right)\;\cdots \;h_{n,L_{n}}\left(t\right)\right]}^{T},\;n=1,2,\ldots ,N$, where the superscript ${}^{T}$ denotes the transpose operator. Furthermore, we assume that $h_{n}\left(t\right),\;n=1,2,\ldots ,N$ are zero-mean random vectors, which follow a simplified first-order Markov model

(1) $$\begin{array}{cc} h_{n}\left(t\right) & =h_{n}\left(t-1\right)+w_{n}\left(t\right), \end{array}$$

where $w_{n}\left(t\right),\;n=1,2,\ldots ,N$ are zero-mean white Gaussian noise vectors [uncorrelated with $h_{n}\left(t-1\right)$], whose correlation matrices are $R_{w_{n}}=\sigma_{w_{n}}^{2}I_{L_{n}},\;n=1,2,\ldots ,N$, with $I_{L_{n}}$ being an identity matrix of size $L_{n}\times L_{n}$. The variances $\sigma_{w_{n}}^{2},\;n=1,2,\ldots ,N$ capture the uncertainties in the corresponding channels $h_{n}\left(t\right),\;n=1,2,\ldots ,N$.

The impulse responses of the channels can be grouped in a tensorial form, i.e., $H\left(t\right)\in R^{L_{1}\times L_{2}\times \cdots \times L_{N}}$, such that

(2) $$\begin{array}{cc} H(t) & =h_{1}\left(t\right)\circ h_{2}\left(t\right)\circ \cdots \circ h_{N}\left(t\right), \end{array}$$

where ∘ denotes the outer product. Therefore, the elements of this rank-1 tensor are ${\left(H\right)}_{l_{1},l_{2},\ldots ,l_{N}}\left(t\right)=h_{1,l_{1}}\left(t\right)h_{2,l_{2}}\left(t\right)\cdots h_{N,l_{N}}\left(t\right)$. In addition, using the vectorization operation, vec(·), we can write

(3) $$\begin{array}{cc} \mathrm{vec}\left[H(t)\right] & =h_{N}\left(t\right)⊗h_{N-1}\left(t\right)⊗\cdots ⊗h_{1}\left(t\right), \end{array}$$

where ⊗ denotes the Kronecker product.

The real-valued input signals that feed into the MISO system are described in the tensorial form $X\left(t\right)\in R^{L_{1}\times L_{2}\times \cdots \times L_{N}}$, having the elements ${\left(X\right)}_{l_{1}l_{2}\ldots l_{N}}\left(t\right)=x_{l_{1}l_{2}\ldots l_{N}}\left(t\right)$. Consequently, the output signal at the discrete-time index *t* results in

(4) $$\begin{array}{cc} y(t) & =X\left(t\right)\times_{1}h_{1}^{T}\left(t\right)\times_{2}h_{2}^{T}\left(t\right)\times_{3}\cdots \times_{N}h_{N}^{T}\left(t\right)\\ \; & =\sum_{l_{1}=1}^{L_{1}}\sum_{l_{2}=1}^{L_{2}}\cdots \sum_{l_{N}=1}^{L_{N}}x_{l_{1}l_{2}\ldots l_{N}}\left(t\right)h_{1,l_{1}}\left(t\right)h_{2,l_{2}}\left(t\right)\cdots h_{N,l_{N}}\left(t\right)\\ \; & ={\mathrm{vec}}^{T}\left[H(t)\right]\mathrm{vec}\left[X(t)\right], \end{array}$$

where $\times_{n}$ denotes the mode-*n* product [5]. This represents a multilinear form, since it is a linear function of each of the vectors $h_{n}\left(t\right),\;n=1,2,\ldots ,N$, considering that the other N−1 components are fixed.

The last line in (4) represents a particular case of the MISO system identification problem, which resembles a single-input, single-output (SISO) scenario. Using the notation $x\left(t\right)=\mathrm{vec}\left[X(t)\right]$ and $h\left(t\right)=\mathrm{vec}\left[H(t)\right]$, the output signal from (4) becomes

(5) $$\begin{array}{cc} y(t) & =h^{T}\left(t\right)x\left(t\right), \end{array}$$

where the vector h(t) represents the global impulse response of the system. Therefore, in this multilinear framework, the system identification problem can be formulated in two ways. First, this can be approached in terms of estimating the individual channels, $h_{n}\left(t\right),\;n=1,2,\ldots ,N$. Alternatively, we can focus on the identification of the global impulse response, h(t), as in a conventional SISO system identification problem.

At this point, there are two important aspects that should be outlined. The global impulse response, h(t), is of length $L=\prod_{n=1}^{N}L_{n}$, but this results as a combination of $\sum_{n=1}^{N}L_{n}$ elements, which are the coefficients of the individual impulse responses, $h_{n}\left(t\right),\;n=1,2,\ldots ,N$. Additionally, according to (3), we have

(6) $$\begin{array}{cc} h(t) & =h_{N}\left(t\right)⊗h_{N-1}\left(t\right)⊗\cdots ⊗h_{1}\left(t\right)\\ \; & =\eta_{N}h_{N}\left(t\right)⊗\eta_{N-1}h_{N-1}\left(t\right)⊗\cdots ⊗\eta_{1}h_{1}\left(t\right), \end{array}$$

with $\eta_{n}\in R$, $\eta_{n}\neq 0$ (for any n=1,2,…,N), and $\prod_{n=1}^{N}\eta_{n}=1$, so that the decomposition of h(t) is not unique. Consequently, from a system identification perspective, it would be more advantageous to approach the problem in terms of estimating the individual impulse responses $h_{n}\left(t\right),\;n=1,2,\ldots ,N$, while the estimated global impulse response results similar to (6). On the other hand, the Kronecker product-based decomposition of h(t) does not lead to a unique set of estimates for the individual channels. However, there is no scaling ambiguity when identifying the global impulse response. In sum, the main idea behind the decomposition-based approach is to reformulate a high-dimension system identification problem as a combination of low-dimension solutions, which are “tensorized” together.

In realistic system identification scenarios, the output signal y(t) is usually corrupted by an additive noise, v(t), which is uncorrelated with the input signals. The variance of this noise signal is $\sigma_{v}^{2}=E\left[v^{2}\left(t\right)\right]$, where E[·] denotes mathematical expectation. Thus, the reference (or desired) signal at the discrete-time index *t* results in

(7) $$\begin{array}{c} d(t)=y(t)+v(t). \end{array}$$

The goal is to estimate the output of the system (or, equivalently, the impulse responses of the channels), given the reference and the input signals. In this context, the conventional Wiener filter provides the well-known solution [20,21,22]

(8) $$\begin{array}{cc} {\hat{h}}_{W} & =R^{-1}p, \end{array}$$

where ${\hat{h}}_{W}$ is an estimate of the global impulse response, while $R=E\left[x\left(t\right)x^{T}\left(t\right)\right]$ and $p=E\left[d(t)x(t)\right]$ represent the covariance matrix and the cross-correlation vector, respectively. Recently, an iterative Wiener filter [38] was developed in the framework of multilinear forms. It exploits the decomposition of the global impulse response, while the optimization criterion is applied, following a block coordinate descent approach, to the individual components [41]. As compared to the conventional Wiener filter from (8), the iterative version of multilinear forms leads to a superior performance, especially when a small amount of data are available for the estimation of R and p. Nevertheless, both previously mentioned Wiener solutions present several limitations, such as the time-invariant framework, the matrix inversion operation, and the estimation of the statistics. These could make them unsuitable in real-world scenarios, e.g., working in nonstationary environments and/or requiring real-time processing.

In this context, a more appropriate approach is adaptive filtering. Since the LMS algorithm represents one of the simplest and most practical solutions, the LMS-based algorithms tailored to the identification of multilinear forms were developed in [39]. Furthermore, the RLS algorithm for multilinear forms was introduced in [40]. These versions are also referred to as tensor-based adaptive algorithms. They rely on the minimization of cost functions that depend on the error signal, i.e., the difference between the reference signal and the estimated output of the system. Nevertheless, in a realistic system identification framework, [i.e., in the presence of the system noise, according to (7)], the goal of the adaptive filter is not to make the error signal reach zero. The objective, instead, is to recover this system noise from the error signal of the adaptive filter, after it converges to the true solution. Consequently, it makes more sense to minimize the system misalignment, i.e., a measure of the difference between the true impulse response of the system and the estimated one. This is the optimization approach behind Kalman filtering. Moreover, the Kalman filter uses a specific parameter that captures the uncertainties in the system to be identified, as outlined in the discussion that follows (1), related to $\sigma_{w_{n}}^{2},\;n=1,2,\ldots ,N$. These parameters could act as control factors. On the other hand, the LMS and RLS adaptive filters do not depend on these uncertainties, since the impulse responses of the channels are considered as deterministic in the derivation of these algorithms. Therefore, the specific parameters of the Kalman filter would allow for better control.

## 3. Kalman Filter for the Identification of Multilinear Forms

Following (5), we can introduce the a priori error signal

(9) $$\begin{array}{cc} e(t) & =d\left(t\right)-\hat{y}\left(t\right)\\ \; & =d\left(t\right)-{\hat{h}}^{T}\left(t-1\right)x\left(t\right), \end{array}$$

where $\hat{y}\left(t\right)$ is the estimated output signal and $\hat{h}\left(t-1\right)$ represents an estimate of the global impulse response at the discrete-time index t−1. Since the global impulse response can be deconstructed based on (6), we can also consider that $\hat{h}\left(t\right)$ similarly results in a combination of the estimated impulse responses of the channels, denoted by ${\hat{h}}_{n}\left(t\right),\;n=1,2,\ldots ,N$. Therefore, we have

(10) $$\begin{array}{cc} \hat{h}\left(t\right) & ={\hat{h}}_{N}\left(t\right)⊗{\hat{h}}_{N-1}\left(t\right)⊗\cdots ⊗{\hat{h}}_{2}\left(t\right)⊗{\hat{h}}_{1}\left(t\right). \end{array}$$

Alternatively, using the properties of the Kronecker product [42], (10) can be expressed in *N* equivalent ways, i.e.,

$$\begin{array}{cc} \hat{h}\left(t\right) & =\left[{\hat{h}}_{N}\left(t\right)⊗{\hat{h}}_{N-1}\left(t\right)⊗\cdots ⊗{\hat{h}}_{2}\left(t\right)⊗I_{L_{1}}\right]{\hat{h}}_{1}\left(t\right)\\ \; & =\left[{\hat{h}}_{N}\left(t\right)⊗{\hat{h}}_{N-1}\left(t\right)⊗\cdots ⊗I_{L_{2}}⊗{\hat{h}}_{1}\left(t\right)\right]{\hat{h}}_{2}\left(t\right)\\ \; & \;\;\vdots \\ \; & =\left[I_{L_{N}}⊗{\hat{h}}_{N-1}\left(t\right)⊗\cdots ⊗{\hat{h}}_{2}\left(t\right)⊗{\hat{h}}_{1}\left(t\right)\right]{\hat{h}}_{N}\left(t\right). \end{array}$$

Consequently, the a priori error signal from (9) can also be rewritten in *N* equivalent forms (targeting the individual filters), as follows

(11) $$\begin{array}{cc} e_{{\hat{h}}_{1}}\left(t\right) & =d\left(t\right)-{\hat{h}}_{1}^{T}\left(t-1\right)x_{{\hat{h}}_{2}{\hat{h}}_{3}\cdots {\hat{h}}_{N}}\left(t\right), \end{array}$$

(12) $$\begin{array}{cc} e_{{\hat{h}}_{2}}\left(t\right) & =d\left(t\right)-{\hat{h}}_{2}^{T}\left(t-1\right)x_{{\hat{h}}_{1}{\hat{h}}_{3}\cdots {\hat{h}}_{N}}\left(t\right),\\ \; & \;\;\vdots \end{array}$$

(13) $$\begin{array}{cc} e_{{\hat{h}}_{N}}\left(t\right) & =d\left(t\right)-{\hat{h}}_{N}^{T}\left(t-1\right)x_{{\hat{h}}_{1}{\hat{h}}_{2}\cdots {\hat{h}}_{N-1}}\left(t\right), \end{array}$$

where

(14) $$\begin{array}{cc} x_{{\hat{h}}_{2}{\hat{h}}_{3}\cdots {\hat{h}}_{N}}\left(t\right) & ={\left[{\hat{h}}_{N}\left(t-1\right)⊗{\hat{h}}_{N-1}\left(t-1\right)⊗\cdots ⊗{\hat{h}}_{2}\left(t-1\right)⊗I_{L_{1}}\right]}^{T}x\left(t\right), \end{array}$$

(15) $$\begin{array}{cc} x_{{\hat{h}}_{1}{\hat{h}}_{3}\cdots {\hat{h}}_{N}}\left(t\right) & ={\left[{\hat{h}}_{N}\left(t-1\right)⊗{\hat{h}}_{N-1}\left(t-1\right)⊗\cdots ⊗I_{L_{2}}⊗{\hat{h}}_{1}\left(t-1\right)\right]}^{T}x\left(t\right),\\ \; & \;\;\vdots \end{array}$$

(16) $$\begin{array}{cc} x_{{\hat{h}}_{1}{\hat{h}}_{2}\cdots {\hat{h}}_{N-1}}\left(t\right) & ={\left[I_{L_{N}}⊗{\hat{h}}_{N-1}\left(t-1\right)⊗\cdots ⊗{\hat{h}}_{2}\left(t-1\right)⊗{\hat{h}}_{1}\left(t-1\right)\right]}^{T}x\left(t\right). \end{array}$$

In a similar manner, the reference signal from (7) can be expressed in *N* equivalent ways, which can be summarized as

(17) $$\begin{array}{cc} d(n) & =h_{n}^{T}\left(t\right)x_{h_{1}h_{2}\cdots h_{n-1}h_{n+1}\cdots h_{N}}\left(t\right)+v\left(n\right), \end{array}$$

where

$$\begin{array}{cc} x_{h_{1}h_{2}\cdots h_{n-1}h_{n+1}\cdots h_{N}}\left(t\right) & =\left[h_{N}\left(t-1\right)⊗h_{N-1}\left(t-1\right)⊗\cdots ⊗h_{n+1}\left(t-1\right)⊗I_{L_{n}}\right.\\ \; & \;\;\;⊗h_{n-1}\left(t-1\right)⊗{\left.\cdots ⊗h_{2}\left(t-1\right)⊗h_{1}\left(t-1\right)\right]}^{T}x\left(t\right), \end{array}$$

with n=1,2,…,N. For any value of n=1,2,…,N, expression (17) plays the role of an observation equation, while (1) represents a state equation. In the framework of multilinear forms, having *N* such pairs of state and observation equations, the objective is to find the optimal recursive estimator of $h_{n}\left(t\right)$, for n=1,2,…,N, i.e., ${\hat{h}}_{n}\left(t\right)$. To this end, we will follow a multilinear optimization approach [41], by considering that N−1 impulse responses are fixed for all the previous time indices, while optimizing the remaining one at the current time index. In this case, within the optimization criterion of ${\hat{h}}_{n}\left(t\right),\;n=1,2,\ldots ,N$, we may assume that $x_{{\hat{h}}_{1}{\hat{h}}_{2}\cdots {\hat{h}}_{n-1}{\hat{h}}_{n+1}\cdots {\hat{h}}_{N}}\left(t\right)\approx x_{h_{1}h_{2}\cdots h_{n-1}h_{n+1}\cdots h_{N}}\left(t\right)$.

Under these considerations and based on (17), we can introduce the a posteriori errors related to the *N* individual filters, which result in

(18) $$\begin{array}{cc} \epsilon_{{\hat{h}}_{n}}\left(t\right) & =d\left(t\right)-{\hat{h}}_{n}^{T}\left(t\right)x_{{\hat{h}}_{1}{\hat{h}}_{2}\cdots {\hat{h}}_{n-1}{\hat{h}}_{n+1}\cdots {\hat{h}}_{N}}\left(t\right)\\ \; & =h_{n}^{T}\left(t\right)x_{h_{1}h_{2}\cdots h_{n-1}h_{n+1}\cdots h_{N}}\left(t\right)+v\left(t\right)-{\hat{h}}_{n}^{T}\left(t\right)x_{{\hat{h}}_{1}{\hat{h}}_{2}\cdots {\hat{h}}_{n-1}{\hat{h}}_{n+1}\cdots {\hat{h}}_{N}}\left(t\right)\\ \; & \approx \left[h_{n}^{T}\left(t\right)-{\hat{h}}_{n}^{T}\left(t\right)\right]x_{{\hat{h}}_{1}{\hat{h}}_{2}\cdots {\hat{h}}_{n-1}{\hat{h}}_{n+1}\cdots {\hat{h}}_{N}}\left(t\right)+v\left(t\right)\\ \; & =\mu_{h_{n}}^{T}\left(t\right)x_{{\hat{h}}_{1}{\hat{h}}_{2}\cdots {\hat{h}}_{n-1}{\hat{h}}_{n+1}\cdots {\hat{h}}_{N}}\left(t\right)+v\left(t\right), \end{array}$$

where $\mu_{h_{n}}\left(t\right)=h_{n}\left(t\right)-{\hat{h}}_{n}\left(t\right)$ is the a posteriori misalignment (or the state estimation error) of the *n*th individual filter, with n=1,2,…,N. Similarly, we can define the a priori misalignments $m_{h_{n}}\left(t\right)=h_{n}\left(t\right)-{\hat{h}}_{n}\left(t-1\right),\;n=1,2,\ldots ,N$, so that [based on (1)]

(19) $$\begin{array}{cc} m_{h_{n}}\left(t\right) & =\mu_{h_{n}}\left(t-1\right)+w_{n}\left(t\right) \end{array}$$

and, consequently,

(20) $$\begin{array}{cc} R_{m_{h_{n}}}\left(t\right) & =R_{\mu_{h_{n}}}\left(t-1\right)+\sigma_{w_{n}}^{2}I_{L_{n}}, \end{array}$$

for n=1,2,…,N, where $R_{m_{h_{n}}}\left(t\right)=E\left[m_{h_{n}}\left(t\right)m_{h_{n}}^{T}\left(t\right)\right]$ and $R_{\mu_{h_{n}}}\left(t\right)=E\left[\mu_{h_{n}}\left(t\right)\mu_{h_{n}}^{T}\left(t\right)\right]$ represent the correlation matrices of the a priori and a posteriori misalignments, respectively.

As explained in Section 2, according to (6), we can only identify the individual impulse responses up to some arbitrary scaling factors, $\eta_{n}$, n=1,2,…,N. However, in terms of identifying the global impulse response (or, equivalently, the output signal), the group $\eta_{1}h_{1}\left(t\right),\eta_{2}h_{2}\left(t\right),\ldots ,\eta_{N}h_{N}\left(t\right)$ is equivalent to the group $h_{1}\left(t\right),h_{2}\left(t\right),\ldots ,h_{N}\left(t\right)$. Thus, in order to simplify the notation, the scaling factors do not appear explicitly in the misalignments.

In the context of the multilinear optimization strategy and the linear sequential Bayesian approach [43], the optimum estimates of the state vectors, ${\hat{h}}_{n}\left(t\right),\;n=1,2,\ldots ,N$, have the recursive forms

(21) $$\begin{array}{cc} {\hat{h}}_{1}\left(t\right) & ={\hat{h}}_{1}\left(t-1\right)+k_{{\hat{h}}_{1}}\left(t\right)\left[d\left(t\right)-{\hat{h}}_{1}^{T}\left(t-1\right)x_{{\hat{h}}_{2}{\hat{h}}_{3}\cdots {\hat{h}}_{N}}\left(t\right)\right]\\ \; & ={\hat{h}}_{1}\left(t-1\right)+k_{{\hat{h}}_{1}}\left(t\right)e\left(t\right), \end{array}$$

(22) $$\begin{array}{cc} {\hat{h}}_{2}\left(t\right) & ={\hat{h}}_{2}\left(t-1\right)+k_{{\hat{h}}_{2}}\left(t\right)\left[d\left(t\right)-{\hat{h}}_{2}^{T}\left(t-1\right)x_{{\hat{h}}_{1}{\hat{h}}_{3}\cdots {\hat{h}}_{N}}\left(t\right)\right]\\ \; & ={\hat{h}}_{2}\left(t-1\right)+k_{{\hat{h}}_{2}}\left(t\right)e\left(t\right),\\ \; & \;\;\vdots \end{array}$$

(23) $$\begin{array}{cc} {\hat{h}}_{N}\left(t\right) & ={\hat{h}}_{N}\left(t-1\right)+k_{{\hat{h}}_{N}}\left(t\right)\left[d\left(t\right)-{\hat{h}}_{N}^{T}\left(t-1\right)x_{{\hat{h}}_{1}{\hat{h}}_{2}\cdots {\hat{h}}_{N-1}}\left(t\right)\right]\\ \; & ={\hat{h}}_{N}\left(t-1\right)+k_{{\hat{h}}_{N}}\left(t\right)e\left(t\right), \end{array}$$

where $k_{{\hat{h}}_{n}}\left(t\right),\;n=1,2,\ldots ,N$ are the so-called Kalman gain vectors, and $e_{{\hat{h}}_{1}}\left(t\right)=e_{{\hat{h}}_{2}}\left(t\right)=\cdots =e_{{\hat{h}}_{N}}\left(t\right)=e\left(t\right)$ [based on (9) and (11)–(13)]. Next, the Kalman gain vectors are obtained by minimizing the cost functions:

(24) $$\begin{array}{cc} J_{{\hat{h}}_{2}{\hat{h}}_{3}\cdots {\hat{h}}_{N}}\left[k_{{\hat{h}}_{1}}\left(t\right)\right] & =\frac{1}{L_{1}}\mathrm{tr}\left[R_{\mu_{h_{1}}}\left(t\right)\right], \end{array}$$

(25) $$\begin{array}{cc} J_{{\hat{h}}_{1}{\hat{h}}_{3}\cdots {\hat{h}}_{N}}\left[k_{{\hat{h}}_{2}}\left(t\right)\right] & =\frac{1}{L_{2}}\mathrm{tr}\left[R_{\mu_{h_{2}}}\left(t\right)\right],\\ \; & \;\;\vdots \end{array}$$

(26) $$\begin{array}{cc} J_{{\hat{h}}_{1}{\hat{h}}_{2}\cdots {\hat{h}}_{N-1}}\left[k_{{\hat{h}}_{N}}\left(t\right)\right] & =\frac{1}{L_{N}}\mathrm{tr}\left[R_{\mu_{h_{N}}}\left(t\right)\right], \end{array}$$

where $\mathrm{tr}\left[\cdot \right]$ denotes the trace of a square matrix. This leads to

(27) $$\begin{array}{cc} k_{{\hat{h}}_{1}}\left(t\right) & =\frac{R_{m_{h_{1}}}\left(t\right)x_{{\hat{h}}_{2}{\hat{h}}_{3}\cdots {\hat{h}}_{N}}\left(t\right)}{x_{{\hat{h}}_{2}{\hat{h}}_{3}\cdots {\hat{h}}_{N}}^{T}\left(t\right)R_{m_{h_{1}}}\left(t\right)x_{{\hat{h}}_{2}{\hat{h}}_{3}\cdots {\hat{h}}_{N}}\left(t\right)+\sigma_{v}^{2}}, \end{array}$$

(28) $$\begin{array}{cc} k_{{\hat{h}}_{2}}\left(t\right) & =\frac{R_{m_{h_{2}}}\left(t\right)x_{{\hat{h}}_{1}{\hat{h}}_{3}\cdots {\hat{h}}_{N}}\left(t\right)}{x_{{\hat{h}}_{1}{\hat{h}}_{3}\cdots {\hat{h}}_{N}}^{T}\left(t\right)R_{m_{h_{2}}}\left(t\right)x_{{\hat{h}}_{1}{\hat{h}}_{3}\cdots {\hat{h}}_{N}}\left(t\right)+\sigma_{v}^{2}},\\ \; & \;\;\vdots \end{array}$$

(29) $$\begin{array}{cc} k_{{\hat{h}}_{N}}\left(t\right) & =\frac{R_{m_{h_{N}}}\left(t\right)x_{{\hat{h}}_{1}{\hat{h}}_{2}\cdots {\hat{h}}_{N-1}}\left(t\right)}{x_{{\hat{h}}_{1}{\hat{h}}_{2}\cdots {\hat{h}}_{N-1}}^{T}\left(t\right)R_{m_{h_{N}}}\left(t\right)x_{{\hat{h}}_{1}{\hat{h}}_{2}\cdots {\hat{h}}_{N-1}}\left(t\right)+\sigma_{v}^{2}}, \end{array}$$

and

(30) $$\begin{array}{cc} R_{\mu_{h_{1}}}\left(t\right) & =\left[I_{L_{1}}-k_{{\hat{h}}_{1}}\left(t\right)x_{{\hat{h}}_{2}{\hat{h}}_{3}\cdots {\hat{h}}_{N}}^{T}\left(t\right)\right]R_{m_{h_{1}}}\left(t\right), \end{array}$$

(31) $$\begin{array}{cc} R_{\mu_{h_{2}}}\left(t\right) & =\left[I_{L_{2}}-k_{{\hat{h}}_{2}}\left(t\right)x_{{\hat{h}}_{1}{\hat{h}}_{3}\cdots {\hat{h}}_{N}}^{T}\left(t\right)\right]R_{m_{h_{2}}}\left(t\right),\\ \; & \;\;\vdots \end{array}$$

(32) $$\begin{array}{cc} R_{\mu_{h_{N}}}\left(t\right) & =\left[I_{L_{N}}-k_{{\hat{h}}_{N}}\left(t\right)x_{{\hat{h}}_{1}{\hat{h}}_{2}\cdots {\hat{h}}_{N-1}}^{T}\left(t\right)\right]R_{m_{h_{N}}}\left(t\right). \end{array}$$

The resulting Kalman filter for multilinear forms is defined by the relations (11)–(13), (20), (27)–(32), followed by the updates (21)–(23), as summarized in Table 1. In order to remain consistent with [40], we will refer to this algorithm as the tensor-based Kalman filter (KF-T).

An estimation of the global impulse response can be obtained based on (10). Alternatively, the conventional Kalman filter can be used to find $\hat{h}\left(t\right)$, based on the observation (7) and a state equation for the global impulse response h(t) [similar to (1)]. In this case, the computational complexity of the conventional Kalman filter would be proportional to $O(L^{2})$, where $L=\prod_{n=1}^{N}L_{n}$ (i.e., the length of the global impulse response, as explained in Section 2). On the other hand, the KF-T algorithm combines the solutions of *N* Kalman-based filters of shorter lengths (i.e., $L_{n},\;n=1,2,\ldots ,N$), so that its computational cost is proportional to $\sum_{n=1}^{N}O\left(L_{n}^{2}\right)$. Moreover, even if they are interconnected, these *N* individual filters can work in parallel, since the update of each filter at the discrete-time index *t* depends on the coefficients of all the other filters from the previous time index, i.e., ${\hat{h}}_{n}\left(t-1\right),\;n=1,2,\ldots ,N$.

## 4. Connection with Tensor-Based Adaptive Filters

The KF-T algorithm developed in the previous section represents a generalization of the Kalman filter for bilinear forms (i.e., the particular case N=2) presented in [27]. Nevertheless, it is also connected with other tensor-based adaptive algorithms, as will be shown in this section.

In [40], the tensor-based RLS (RLS-T) algorithm was introduced in the context of multilinear forms. At first, the RLS-T algorithm with the forgetting factors equal to one [44] has a striking resemblance to the KF-T using $\sigma_{w_{n}}^{2}=0,\;n=1,2,\ldots ,N$. However, the KF-T does not rely on any matrix inversion, which is not the case for the RLS-T algorithm. Moreover, the RLS-T depends on the correlation matrices of the input signals, while the KF-T is related to the correlation matrices of the misalignments. This is a more proper approach in system identification scenarios, as was outlined at the end of Section 2. Additionally, the RLS-T algorithm does not depend on the variance of the additive noise (i.e., the parameter $\sigma_{v}^{2}$), or on the uncertainties in the system (i.e., the parameters $\sigma_{w_{n}}^{2},\;n=1,2,\ldots ,N$), since $h_{n}\left(t\right),\;n=1,2,\ldots ,N$ are considered as deterministic in its derivation. Nevertheless, these specific parameters of the KF-T allow for better control of the algorithm. For example, large values of $\sigma_{w_{n}}^{2}$, n=1,2,…,N are suitable when the uncertainties in the system are high, in which case a good tracking behavior of the algorithm is needed; usually, the price is a lower accuracy, i.e., a higher misalignment. On the other hand, lower values of these parameters lead to improved accuracy, but reduce the tracking capability.

An interesting connection can be established between the KF-T and the tensor-based NLMS (NLMS-T) algorithm [39,40], which was also developed in the framework of multilinear forms. Let us consider that the KF-T has started to converge. Consequently, in the steady-state of the algorithm, we may also consider that $R_{m_{h_{n}}}\left(t\right),\;n=1,2,\ldots ,N$ tend to become diagonal matrices. This approximation is reasonable, taking into account that the misalignment of the individual coefficients tend to become uncorrelated. Hence, we can use

(33) $$\begin{array}{c} R_{m_{h_{n}}}\left(t\right)\approx \sigma_{m_{h_{n}}}^{2}\left(t\right)I_{L_{n}}, \end{array}$$

where $\sigma_{m_{h_{n}}}^{2}\left(t\right),\;n=1,2,\ldots ,N$ represent the elements from the main diagonal of the respective matrices. Furthermore, using the notation

(34) $$\begin{array}{cc} \delta_{h_{n}}\left(t\right) & =\frac{\sigma_{v}^{2}}{\sigma_{m_{h_{n}}}^{2}\left(t\right)},\;n=1,2,\ldots ,N, \end{array}$$

the expressions of the Kalman gain vectors from (27)–(29) simplify to

(35) $$\begin{array}{cc} {\tilde{k}}_{{\hat{h}}_{1}}\left(t\right) & =\frac{x_{{\hat{h}}_{2}{\hat{h}}_{3}\cdots {\hat{h}}_{N}}\left(t\right)}{x_{{\hat{h}}_{2}{\hat{h}}_{3}\cdots {\hat{h}}_{N}}^{T}\left(t\right)x_{{\hat{h}}_{2}{\hat{h}}_{3}\cdots {\hat{h}}_{N}}\left(t\right)+\delta_{h_{1}}\left(t\right)}, \end{array}$$

(36) $$\begin{array}{cc} {\tilde{k}}_{{\hat{h}}_{2}}\left(t\right) & =\frac{x_{{\hat{h}}_{1}{\hat{h}}_{3}\cdots {\hat{h}}_{N}}\left(t\right)}{x_{{\hat{h}}_{1}{\hat{h}}_{3}\cdots {\hat{h}}_{N}}^{T}\left(t\right)x_{{\hat{h}}_{1}{\hat{h}}_{3}\cdots {\hat{h}}_{N}}\left(t\right)+\delta_{h_{2}}\left(t\right)},\\ \; & \;\;\vdots \end{array}$$

(37) $$\begin{array}{cc} {\tilde{k}}_{{\hat{h}}_{N}}\left(t\right) & =\frac{x_{{\hat{h}}_{1}{\hat{h}}_{2}\cdots {\hat{h}}_{N-1}}\left(t\right)}{x_{{\hat{h}}_{1}{\hat{h}}_{2}\cdots {\hat{h}}_{N-1}}^{T}\left(t\right)x_{{\hat{h}}_{1}{\hat{h}}_{2}\cdots {\hat{h}}_{N-1}}\left(t\right)+\delta_{h_{N}}\left(t\right)}, \end{array}$$

so that the updates (21)–(23) become

(38) $$\begin{array}{cc} {\hat{h}}_{1}\left(t\right) & ={\hat{h}}_{1}\left(t-1\right)+{\tilde{k}}_{{\hat{h}}_{1}}\left(t\right)e\left(t\right)\\ \; & ={\hat{h}}_{1}\left(t-1\right)+\frac{x_{{\hat{h}}_{2}{\hat{h}}_{3}\cdots {\hat{h}}_{N}}\left(t\right)e\left(t\right)}{x_{{\hat{h}}_{2}{\hat{h}}_{3}\cdots {\hat{h}}_{N}}^{T}\left(t\right)x_{{\hat{h}}_{2}{\hat{h}}_{3}\cdots {\hat{h}}_{N}}\left(t\right)+\delta_{h_{1}}\left(t\right)}, \end{array}$$

(39) $$\begin{array}{cc} {\hat{h}}_{2}\left(t\right) & ={\hat{h}}_{2}\left(t-1\right)+{\tilde{k}}_{{\hat{h}}_{2}}\left(t\right)e\left(t\right)\\ \; & ={\hat{h}}_{2}\left(t-1\right)+\frac{x_{{\hat{h}}_{1}{\hat{h}}_{3}\cdots {\hat{h}}_{N}}\left(t\right)e\left(t\right)}{x_{{\hat{h}}_{1}{\hat{h}}_{3}\cdots {\hat{h}}_{N}}^{T}\left(t\right)x_{{\hat{h}}_{1}{\hat{h}}_{3}\cdots {\hat{h}}_{N}}\left(t\right)+\delta_{h_{2}}\left(t\right)},\\ \; & \;\;\vdots \end{array}$$

(40) $$\begin{array}{cc} {\hat{h}}_{N}\left(t\right) & ={\hat{h}}_{N}\left(t-1\right)+{\tilde{k}}_{{\hat{h}}_{N}}\left(t\right)e\left(t\right)\\ \; & ={\hat{h}}_{N}\left(t-1\right)+\frac{x_{{\hat{h}}_{1}{\hat{h}}_{2}\cdots {\hat{h}}_{N-1}}\left(t\right)e\left(t\right)}{x_{{\hat{h}}_{1}{\hat{h}}_{2}\cdots {\hat{h}}_{N-1}}^{T}\left(t\right)x_{{\hat{h}}_{1}{\hat{h}}_{2}\cdots {\hat{h}}_{N-1}}\left(t\right)+\delta_{h_{N}}\left(t\right)}. \end{array}$$

These updates are specific to a tensor-based variable-regularized NLMS algorithm tailored for multilinear forms, namely VR-NLMS-T. Such an algorithm is defined by the error signals from (11)–(13) and the updates (38)–(40). In this case, the control parameters are grouped into the variable regularization factors $\delta_{h_{n}}\left(t\right),\;n=1,2,\ldots ,N$.

The VR-NLMS-T algorithm represents a simplified form of the KF-T. Nevertheless, due to the assumptions (33), the convergence features of the KF-T outperform the VR-NLMS-T algorithm. Starting from the VR-NLMS-T algorithm, we can show the connections with other tensor-based algorithms. For example, using constant values for the regularization parameters in the denominators of (38)–(40), i.e., $\delta_{h_{n}}\left(t\right)=\delta_{n},\;n=1,2,\ldots ,N$, while multiplying them with some normalized step-sizes $0<\alpha_{n}\leq 1$ (n=1,2,…,N) the nominators in (38)–(40), we obtain the NLMS-T algorithm [39,40]. Furthermore, replacing $x_{{\hat{h}}_{1}{\hat{h}}_{2}\cdots {\hat{h}}_{n-1}{\hat{h}}_{n+1}\cdots {\hat{h}}_{N}}^{T}\left(t\right)x_{{\hat{h}}_{1}{\hat{h}}_{2}\cdots {\hat{h}}_{n-1}{\hat{h}}_{n+1}\cdots {\hat{h}}_{N}}\left(t\right),\;n=1,2,\ldots ,N$ by $\sum_{n=1}^{N}x_{{\hat{h}}_{1}{\hat{h}}_{2}\cdots {\hat{h}}_{n-1}{\hat{h}}_{n+1}\cdots {\hat{h}}_{N}}^{T}\left(t\right)x_{{\hat{h}}_{1}{\hat{h}}_{2}\cdots {\hat{h}}_{n-1}{\hat{h}}_{n+1}\cdots {\hat{h}}_{N}}\left(t\right)$, the tensor LMS algorithm [23] is obtained.

## 5. Simulation Results

In this section, simulation results are provided in order to support the performance of the proposed KF-T and the main theoretical findings related to this algorithm. The goal of this analysis is fourfold, as follows. First, we evaluate the influence of the uncertainty parameters (i.e., $\sigma_{w_{n}}^{2},\;n=1,2,\ldots ,N$) on the performance of the KF-T. Second, we outline the connection between the proposed Kalman-based algorithm and its tensor-based counterparts, as discussed in Section 4. Third, we assess the performance of the KF-T as compared to the conventional Kalman filter. Finally, we analyze the behavior of the KF-T in a more general framework, for the identification of nonseparable systems.

The experiments are performed in the context of the multilinear framework presented in Section 2. The order of the system is N=4 and the input signals that form the tensor X(t) are AR(1) processes in most of the experiments. These inputs are obtained by filtering white Gaussian noises through an AR(1) model with a pole at 0.99. Such a high correlation degree (due to the pole close to 1) represents a challenge for most adaptive filters [20,21,22], in terms of their convergence features. In the last two experiments, we also used real-world speech sequences (corrupted by background noise) as input signals, which are also challenging due to their nonstationary character.

The MISO system used in simulations is characterized by four individual channels (i.e., N=4), where their impulse responses are chosen as follows. The impulse response $h_{1}\left(t\right)$ contains the first $L_{1}=16$ coefficients of the first network echo path from the ITU-T G168 Recommendation [45] (which is a standard for digital network echo cancellers). The impulse response $h_{2}\left(t\right)$ is randomly generated (with Gaussian distribution), using the length $L_{2}=8$. The lengths of the other two impulse responses, i.e., $h_{3}\left(t\right)$ and $h_{4}\left(t\right)$, are set to $L_{3}=4$ and $L_{4}=2$, respectively. Their coefficients follow an exponential decay based on the rule $a_{k}^{l_{k}-1},\;l_{k}=1,\ldots ,L_{k},\;k=3,4$, using $a_{3}=0.8$ and $a_{4}=0.3$, respectively. The length of the global impulse response h(t) is $L=\prod_{n=1}^{N}L_{n}$, which, in our case, results in L=1024. Such a length could be prohibiting in terms of implementation, especially for the Kalman-based and RLS-based adaptive filters. On the other hand, the tensor-based algorithms combine the solutions of *N* shorter filters of length $L_{n},\;n=1,2,\ldots ,N$, which is much more advantageous, since, usually, $L_{n}\ll L$ (as in the current setup).

The output signal y(n) from (4) is corrupted by an additive white Gaussian noise, v(n); its variance is set to $\sigma_{v}^{2}=0.01$. We assume that this parameter is available in simulations. In practice, it can be estimated in several ways, e.g., [46,47]. Nevertheless, the influence of these different estimates on the performance of the proposed KF-T is beyond the scope of this paper. Finally, the reference signal results based on (7).

Two performance measures are used in simulations. First, the identification of the global impulse response is evaluated based on the normalized misalignment (NM), in dB, which is computed as

(41) $$\begin{array}{cc} \mathrm{NM}\left[h\left(t\right),\hat{h}\left(t\right)\right] & =10\log_{10}\left[\frac{{∥h\left(t\right)-\hat{h}\left(t\right)∥}^{2}}{{∥h(t)∥}^{2}}\right], \end{array}$$

where $∥\cdot ∥$ stands for the Euclidean norm. Second, since the identification of the individual impulse responses is influenced by the scaling factors [according to the discussion related to (6)], a proper performance measure is the normalized projection misalignment (NPM) [48], which is evaluated as

(42) $$\begin{array}{cc} \mathrm{NPM}\left[h_{n}\left(t\right),{\hat{h}}_{n}\left(t\right)\right] & =10\log_{10}\left\{1-{\left[\frac{h_{n}^{T}\left(t\right){\hat{h}}_{n}\left(t\right)}{∥h_{n}\left(t\right)∥∥{\hat{h}}_{n}\left(t\right)∥}\right]}^{2}\right\}, \end{array}$$

for n=1,2,…,N.

**Figure 1:**
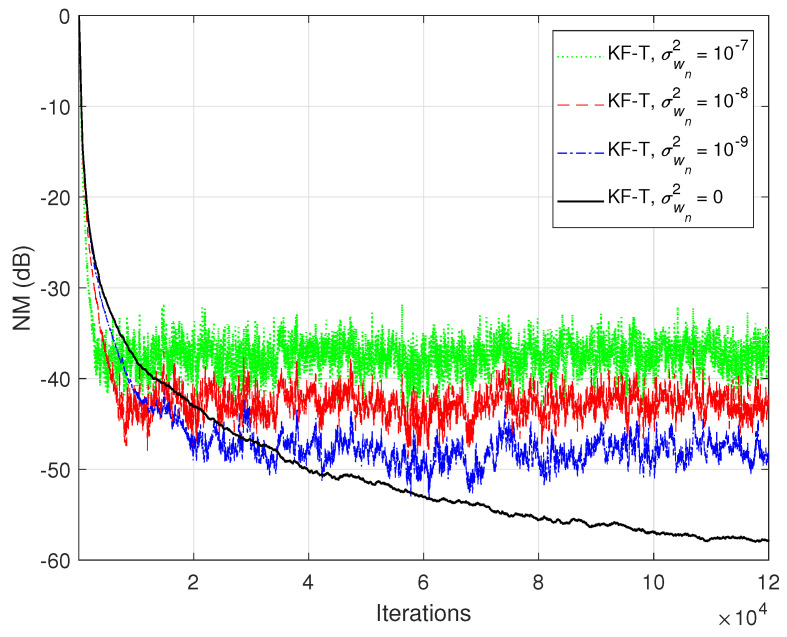
Normalized misalignment (NM) of the KF-T using different values of $\sigma_{w_{n}}^{2},\;n=1,2,\ldots ,N$, for the identification of the global impulse response, h(t).

**Figure 2:**
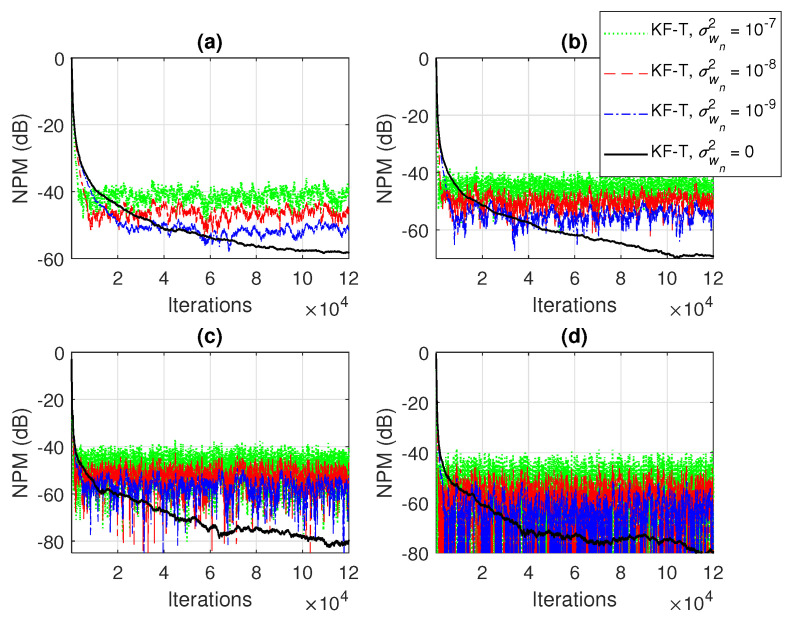
Normalized projection misalignment (NPM) of the KF-T using different values of $\sigma_{w_{n}}^{2},\;n=1,2,\ldots ,N$, for the identification of the individual impulse responses, $h_{n}\left(t\right),\;n=1,2,\ldots ,N$. (**a**) $\mathrm{NPM}\left[h_{1}\left(t\right),{\hat{h}}_{1}\left(t\right)\right]$, (**b**) $\mathrm{NPM}\left[h_{2}\left(t\right),{\hat{h}}_{2}\left(t\right)\right]$, (**c**) $\mathrm{NPM}\left[h_{3}\left(t\right),{\hat{h}}_{3}\left(t\right)\right]$, and (**d**) $\mathrm{NPM}\left[h_{4}\left(t\right),{\hat{h}}_{4}\left(t\right)\right]$.

**Figure 3:**
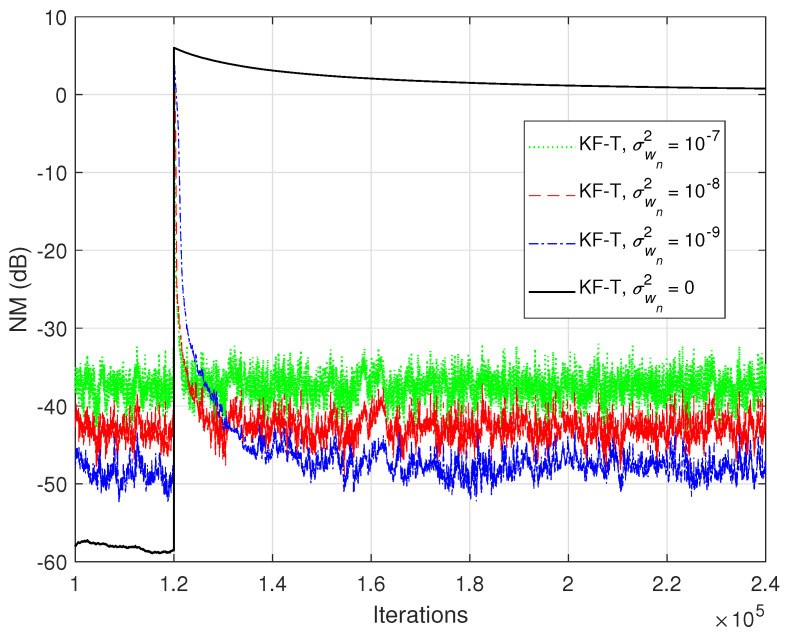
Normalized misalignment (NM) of the KF-T using different values of $\sigma_{w_{n}}^{2},\;n=1,2,\ldots ,N$, for the identification of the global impulse response, h(t). The first individual impulse response changes from $h_{1}\left(t\right)$ to $-h_{1}\left(t\right)$ in the middle of simulation.

In the first set of experiments, we evaluate the impact of the uncertainty parameters on the performance of the proposed KF-T. The values of $\sigma_{w_{n}}^{2},\;n=1,2,\ldots ,N$ are subject to a compromise between the main performance criteria, i.e., fast convergence/tracking versus low misadjustment (i.e., good accuracy). The best accuracy of the solution is obtained for $\sigma_{w_{n}}^{2}=0$, n=1,2,…,N, i.e., when there are no uncertainties in the system. Such a setup is suitable in stationary environments. As can be seen in Figure 1 and Figure 2 (in terms of the NM and NPM, respectively), the lower the values of $\sigma_{w_{n}}^{2},\;n=1,2,\ldots ,N$, the lower the misalignment of KF-T. On the other hand, larger values of these parameters improve the tracking capability of the algorithm, but achieve a higher misadjustment, reducing the accuracy of the solution. This behavior is supported in Figure 3, where an abrupt change in the system is considered in the middle of the experiments, by changing the sign of the coefficients of $h_{1}\left(t\right)$. Since the initial convergence rate is not relevant in this case, and for a better visualization, we focused only on the tracking behavior in Figure 3. Despite having the lowest misalignment (i.e., the best accuracy), the KF-T using $\sigma_{w_{n}}^{2}=0$, n=1,2,…,N has the slowest tracking capability. Nevertheless, larger values of $\sigma_{w_{n}}^{2},\;n=1,2,\ldots ,N$ lead to a significantly better performance in terms of tracking, while slightly sacrificing the accuracy of the solution (i.e., achieving a slightly higher misalignment level).

**Figure 4:**
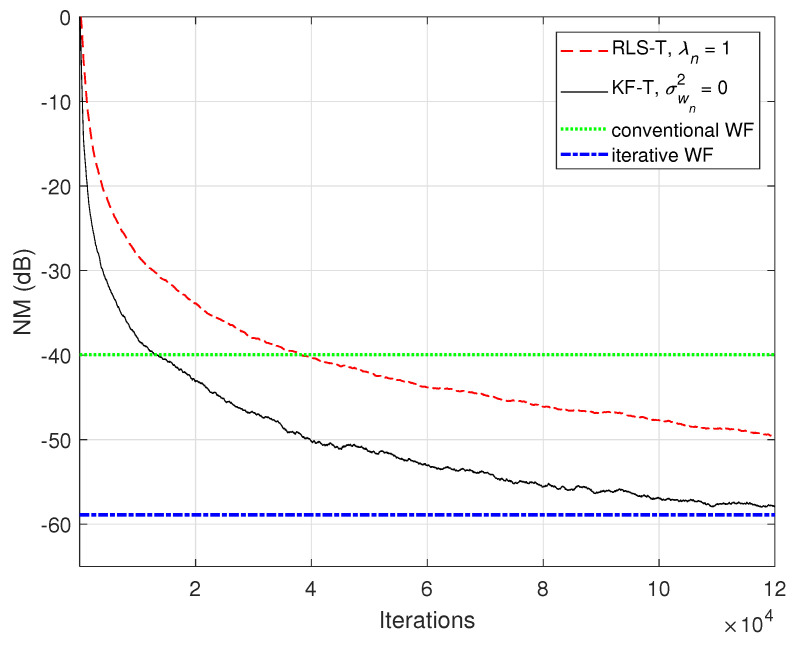
Normalized misalignment (NM) of the RLS-T algorithm using $\lambda_{n}=1,\;n=1,2,\ldots ,N$, the KF-T using $\sigma_{w_{n}}^{2}=0,\;n=1,2,\ldots ,N$, and the conventional and iterative WFs [38], for the identification of the global impulse response, h(t).

**Figure 5:**
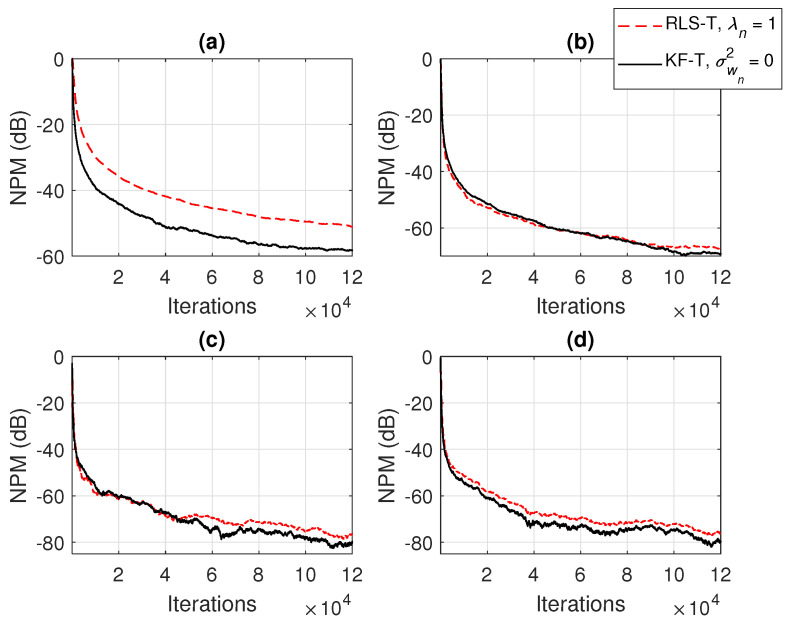
Normalized projection misalignment (NPM) of the RLS-T algorithm using $\lambda_{n}=1,\;n=1,2,\ldots ,N$ and the KF-T using $\sigma_{w_{n}}^{2}=0,\;n=1,2,\ldots ,N$, for the identification of the individual impulse responses, $h_{n}\left(t\right),\;n=1,2,\ldots ,N$. (**a**) $\mathrm{NPM}\left[h_{1}\left(t\right),{\hat{h}}_{1}\left(t\right)\right]$, (**b**) $\mathrm{NPM}\left[h_{2}\left(t\right),{\hat{h}}_{2}\left(t\right)\right]$, (**c**) $\mathrm{NPM}\left[h_{3}\left(t\right),{\hat{h}}_{3}\left(t\right)\right]$, and (**d**) $\mathrm{NPM}\left[h_{4}\left(t\right),{\hat{h}}_{4}\left(t\right)\right]$.

The connections between the proposed KF-T and other tensor-based adaptive algorithms were shown in Section 4. For example, the KF-T with $\sigma_{w_{n}}^{2}=0$, n=1,2,…,N resembles the RLS-T algorithm [40] using the forgetting factors $\lambda_{n}=1,\;n=1,2,\ldots ,N$. This aspect is supported in Figure 4 and Figure 5, in terms of the NM and NPM, respectively. Both algorithms achieve similar initial convergence rates. However, the KF-T reaches a lower misalignment level and outperforms the RLS-T algorithm, thus supporting the discussion from Section 4. Moreover, in Figure 4, we also introduce comparisons with the solutions provided by the conventional and iterative Wiener filters (WFs) [38]. Both tensor-based algorithms outperform the conventional WF in terms of the accuracy of their solutions (i.e., lower misalignment). The KF-T converges (faster than the RLS-T) to the solution obtained by the iterative WF for multilinear forms [38]. Nevertheless, as mentioned in Section 2 [in the discussion that follows (8)], the KF-T overcomes the inherent limitations of the iterative WF (e.g., time-invariant framework, estimation of the statistics, and the matrix inversion operation).

**Figure 6:**
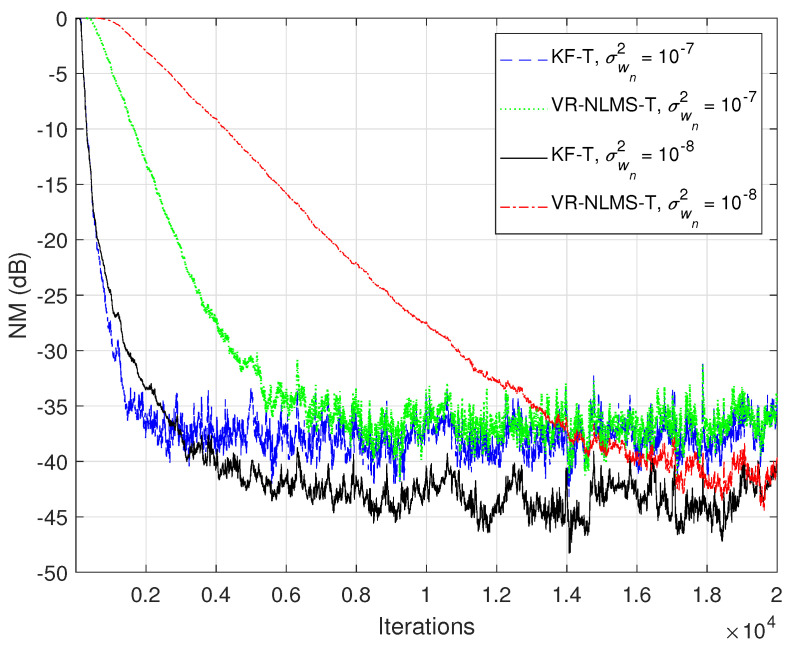
Normalized misalignment (NM) of the KF-T and VR-NLMS-T algorithm (using different values of $\sigma_{w_{n}}^{2},\;n=1,2,\ldots ,N$), for the identification of the global impulse response, h(t).

**Figure 7:**
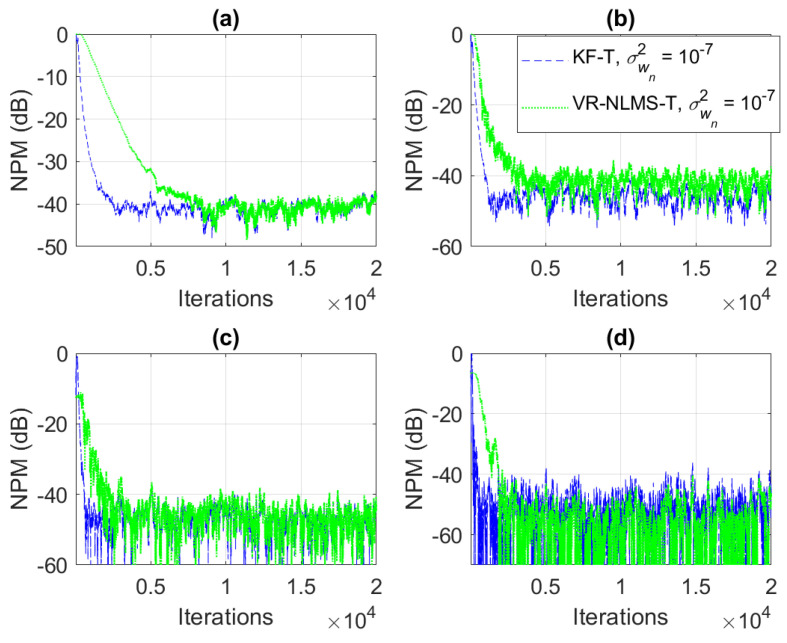
Normalized projection misalignment (NPM) of the KF-T and VR-NLMS-T algorithm using $\sigma_{w_{n}}^{2}=10^{-7},\;n=1,2,\ldots ,N$, for the identification of the individual impulse responses, $h_{n}\left(t\right),\;n=1,2,\ldots ,n$. (**a**) $\mathrm{NPM}\left[h_{1}\left(t\right),{\hat{h}}_{1}\left(t\right)\right]$, (**b**) $\mathrm{NPM}\left[h_{2}\left(t\right),{\hat{h}}_{2}\left(t\right)\right]$, (**c**) $\mathrm{NPM}\left[h_{3}\left(t\right),{\hat{h}}_{3}\left(t\right)\right]$, and (**d**) $\mathrm{NPM}\left[h_{4}\left(t\right),{\hat{h}}_{4}\left(t\right)\right]$.

**Figure 8:**
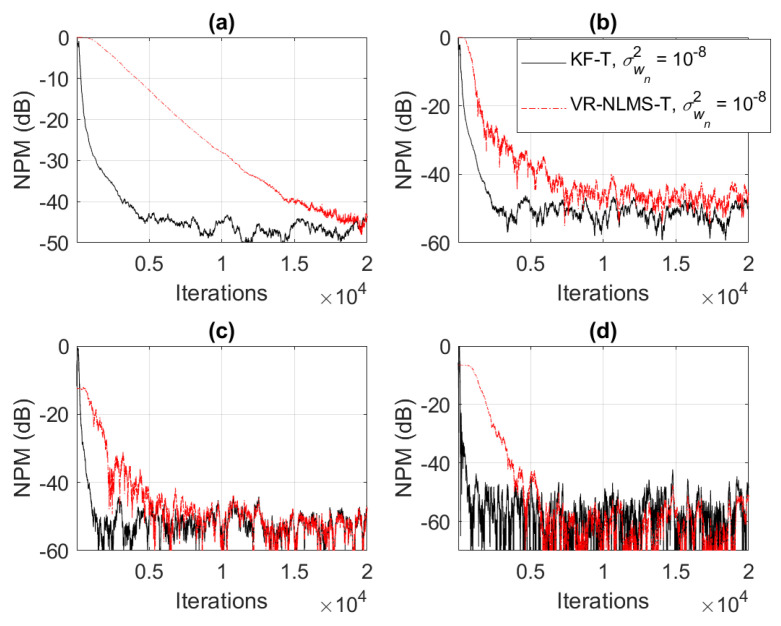
Normalized projection misalignment (NPM) of the KF-T and VR-NLMS-T algorithm using $\sigma_{w_{n}}^{2}=10^{-8},\;n=1,2,\ldots ,N$, for the identification of the individual impulse responses, $h_{n}\left(t\right),\;n=1,2,\ldots ,n$. (**a**) $\mathrm{NPM}\left[h_{1}\left(t\right),{\hat{h}}_{1}\left(t\right)\right]$, (**b**) $\mathrm{NPM}\left[h_{2}\left(t\right),{\hat{h}}_{2}\left(t\right)\right]$, (**c**) $\mathrm{NPM}\left[h_{3}\left(t\right),{\hat{h}}_{3}\left(t\right)\right]$, and (**d**) $\mathrm{NPM}\left[h_{4}\left(t\right),{\hat{h}}_{4}\left(t\right)\right]$.

Based on the assumption from (33), it was also shown in Section 4 that the KF-T behaves like a VR-NLMS-T algorithm, which is defined by the relations (34)–(40). This behavior is supported in Figure 6, Figure 7 and Figure 8, when using different values of $\sigma_{w_{n}}^{2},\;n=1,2,\ldots ,N$. The KF-T and the VR-NLMS-T reach the same misalignment level for the same values of the uncertainty parameters. On the other hand, due to the assumption in (33), the VR-NLMS-T algorithm experiences a slower convergence rate as compared to the KF-T. In fact, as outlined in Section 4, the VR-NLMS-T algorithm represents a simplified version of the KF-T, with a lower computational complexity (but paying a price in terms of the convergence features).

**Figure 9:**
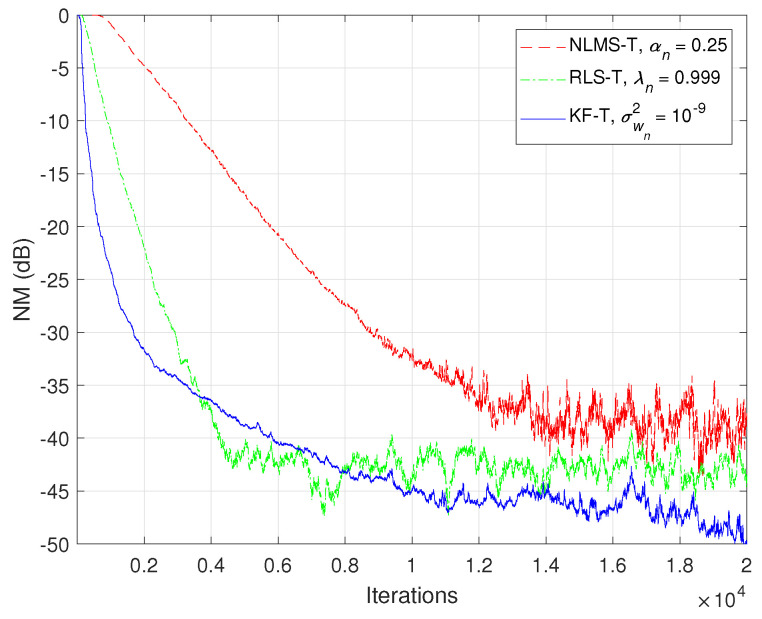
Normalized misalignment (NM) of the NLMS-T algorithm using $\alpha_{n}=0.25,\;n=1,2,\ldots ,N$, the RLS-T algorithm using $\lambda_{n}=0.999,\;n=1,2,\ldots ,N$, and the KF-T using $\sigma_{w_{n}}^{2}=10^{-9},\;n=1,2,\ldots ,N$, for the identification of the global impulse response, h(t).

**Figure 10:**
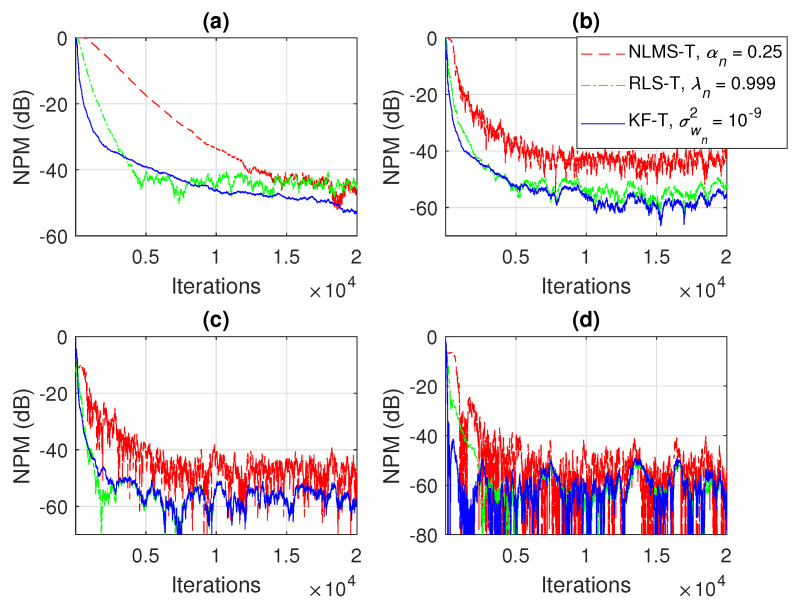
Normalized projection misalignment (NPM) of the NLMS-T algorithm using $\alpha_{n}=0.25,\;n=1,2,\ldots ,N$, the RLS-T algorithm using $\lambda_{n}=0.999,\;n=1,2,\ldots ,N$, and the KF-T using $\sigma_{w_{n}}^{2}=10^{-9},\;n=1,2,\ldots ,N$, for the identification of the individual impulse responses, $h_{n}\left(t\right),\;n=1,2,\ldots ,n$. (**a**) $\mathrm{NPM}\left[h_{1}\left(t\right),{\hat{h}}_{1}\left(t\right)\right]$, (**b**) $\mathrm{NPM}\left[h_{2}\left(t\right),{\hat{h}}_{2}\left(t\right)\right]$, (**c**) $\mathrm{NPM}\left[h_{3}\left(t\right),{\hat{h}}_{3}\left(t\right)\right]$, and (**d**) $\mathrm{NPM}\left[h_{4}\left(t\right),{\hat{h}}_{4}\left(t\right)\right]$.

In Figure 9 and Figure 10, the NM and NPM performance of the proposed KF-T are compared to its tensor-based counterparts, i.e., the NLMS-T and the RLS-T algorithms [40]. The specific parameters of the KF-T are set to $\sigma_{w_{n}}^{2}=10^{-9}$, n=1,2,…,N, while the RLS-T algorithm uses the forgetting factors $\lambda_{n}=0.999,\;n=1,2,\ldots ,N$, in order to target a similar convergence behavior. The normalized step-sizes of the NLMS-T algorithm are set to $\alpha_{n}=0.25$, n=1,2,…,N, which represent the fastest convergence mode [39,40]. The NLMS-T and RLS-T algorithms reach similar misalignment levels, while the KF-T outperforms its counterparts in terms of both convergence rate and misalignment. As expected, the RLS-T is significantly faster (in terms of the convergence rate) as compared to the NLMS-T algorithm. Nevertheless, the KF-T provides an initial convergence rate that is slightly better as compared to the RLS-T algorithm, while also achieving a lower misalignment level (i.e., a better accuracy of the solution).

**Figure 11:**
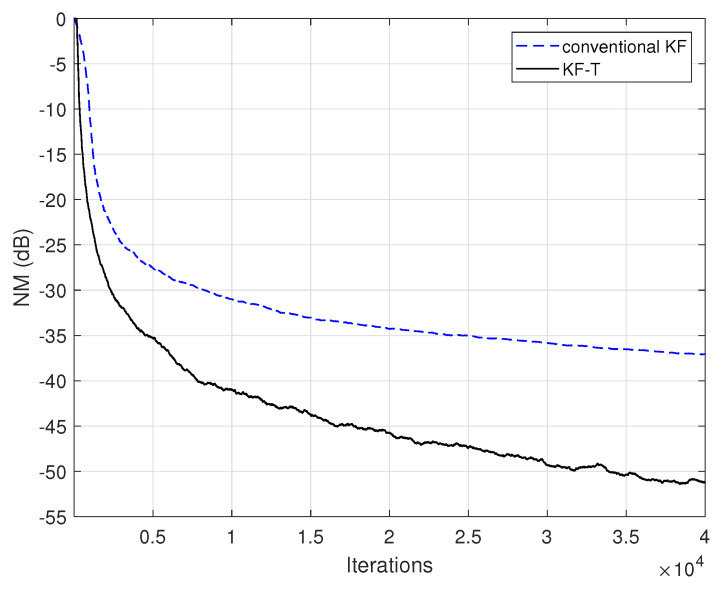
Normalized misalignment (NM) of the KF-T using $\sigma_{w_{n}}^{2}=0,\;n=1,2,\ldots ,N$ and the conventional KF using the same value of its uncertainty parameter, for the identification of the global impulse response, h(t).

The conventional Kalman filter (KF) can also be used for the identification of the global impulse response, h(t), as explained in the end of Section 3. In this case, there is a single adaptive filter (of length *L*) that has to be updated, while the overall computational complexity is proportional to $O(L^{2})$. Due to the large number of coefficients, dealing with such a long adaptive filter raises significant challenges in terms of the complexity, convergence, and accuracy of the solution. On the other hand, the proposed KF-T can obtain the estimate of the global impulse response by combining the solutions of much shorter adaptive filters of lengths $L_{n}$, n=1,2,…,N, with $\prod_{n=1}^{N}L_{n}=L$. Therefore, the expected gain is twofold, in terms of both performance and complexity. This is supported in Figure 11, where the performance of the proposed KF-T is compared to the conventional KF. The specific parameters are set to target the best accuracy of the solution, i.e., $\sigma_{w_{n}}^{2}=0$, n=1,2,…,N in case of the KF-T, while the conventional KF uses the same null value for its uncertainty parameter. The proposed KF-T outperforms the conventional KF in terms of accuracy, achieving a significantly lower misalignment level. Moreover, the complexity of the KF-T is proportional to $\sum_{n=1}^{N}O\left(L_{n}^{2}\right)$, which is much more advantageous compared to the conventional KF, especially for $L_{n}\ll L$, with n=1,2,…,N. For example, related to the experiment given in Figure 11, we could mention that the simulation time (using MATLAB R2018b) of the proposed KF-T was less than one minute, while the conventional KF took almost one hour to reach the final result. The experiment was performed on an Asus GL552VX device (Windows 10 OS), having an Intel Core i7-6700HQ CPU@2.60 GHz, with 4 Cores, 8 Logical Processors, and 16 GB of RAM.

**Figure 12:**
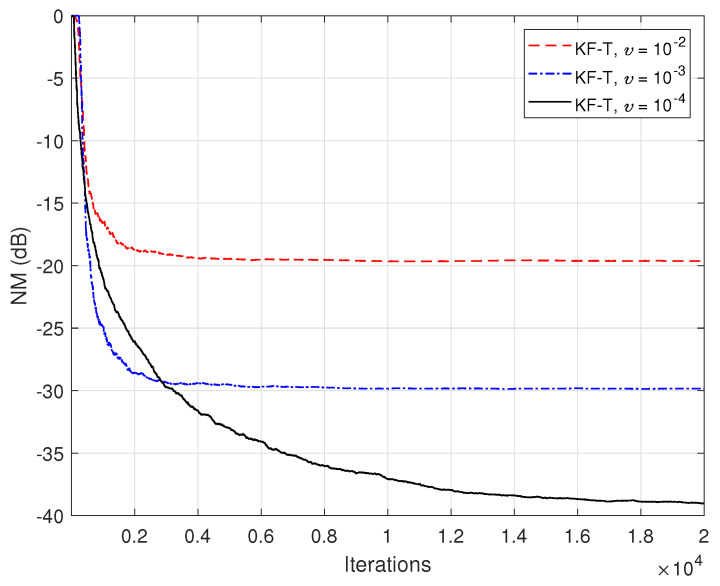
Normalized misalignment (NM) of the KF-T using $\sigma_{w_{n}}^{2}=0,\;n=1,2,\ldots ,N$ for the identification of the global impulse response, h(t)+u(t), where u(t) is randomly generated (Gaussian distribution), with variance $\upsilon {∥h(t)∥}^{2}/L$, using different values of υ.

**Figure 13:**
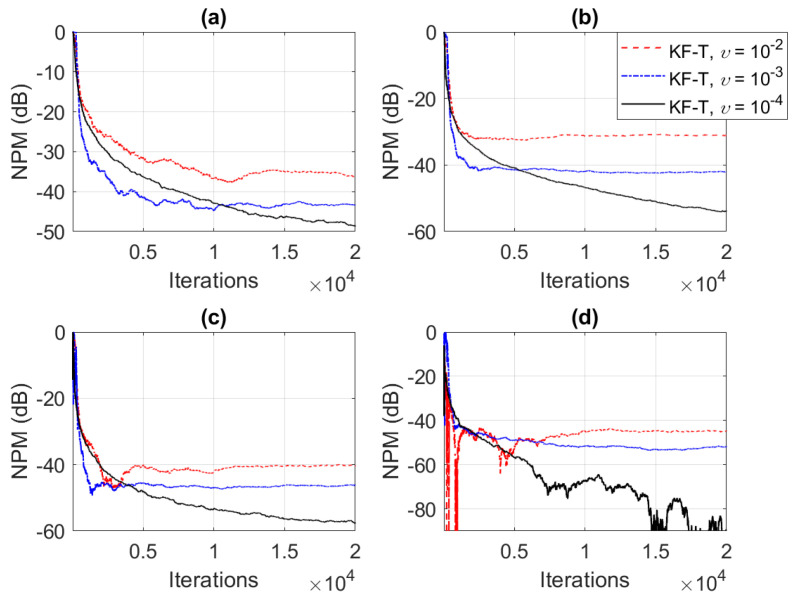
Normalized projection misalignment (NPM) of the KF-T using $\sigma_{w_{n}}^{2}=0,\;n=1,2,\ldots ,N$ for the identification of the individual impulse responses, $h_{n}\left(t\right),\;n=1,2,\ldots ,N$, which result from the decomposition of h(t)+u(t), where u(t) is randomly generated (Gaussian distribution), with variance $\upsilon {∥h(t)∥}^{2}/L$, using different values of υ. (**a**) $\mathrm{NPM}\left[h_{1}\left(t\right),{\hat{h}}_{1}\left(t\right)\right]$, (**b**) $\mathrm{NPM}\left[h_{2}\left(t\right),{\hat{h}}_{2}\left(t\right)\right]$, (**c**) $\mathrm{NPM}\left[h_{3}\left(t\right),{\hat{h}}_{3}\left(t\right)\right]$, and (**d**) $\mathrm{NPM}\left[h_{4}\left(t\right),{\hat{h}}_{4}\left(t\right)\right]$.

In the last set of experiments, we focus on a more challenging scenario, when the global impulse response of the system is not separable. In this case, we target the identification of h(t)+u(t), where h(t) is specified in the beginning of this section [i.e., $h\left(t\right)=h_{4}\left(t\right)⊗h_{3}\left(t\right)⊗h_{2}\left(t\right)⊗h_{1}\left(t\right)$], while u(t) is randomly generated, with Gaussian distribution, and its variance is set to $\upsilon {∥h(t)∥}^{2}/L$ (using different values of υ). Clearly, the higher the value of υ, the more challenging the decomposition of the global impulse response. Since the KF-T is based on the decomposition in (10), it cannot model the noisy part u(t). Nevertheless, as can be seen in Figure 12 and Figure 13 (in terms of the NM and NPM, respectively), the KF-T is able to achieve a reasonable attenuation of the misalignment (i.e., a good accuracy of the estimate) even for larger values of υ. Consequently, the proposed KF-T has uses beyond the identification of rank-1 tensors, when the global impulse response contains a dominant separable (i.e., decomposable) part.

**Figure 14:**
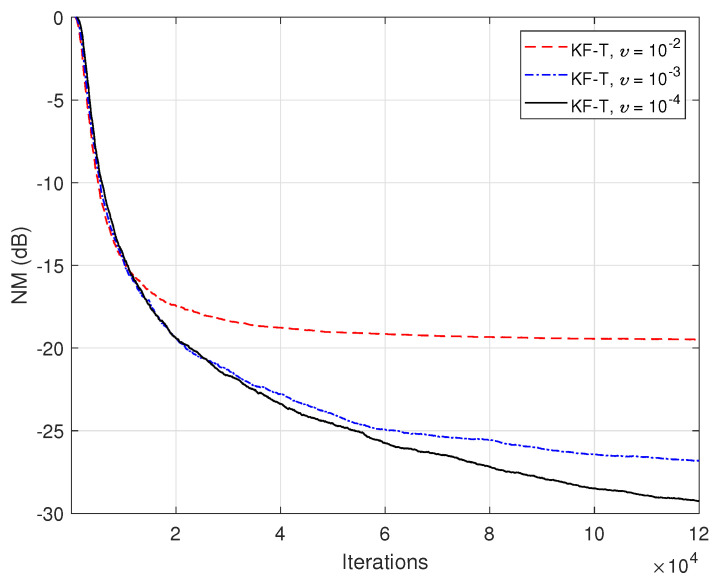
Normalized misalignment (NM) of the KF-T using $\sigma_{w_{n}}^{2}=0,\;n=1,2,\ldots ,N$ for the identification of the global impulse response, h(t)+u(t), where u(t) is randomly generated (Gaussian distribution), with variance $\upsilon {∥h(t)∥}^{2}/L$, using different values of υ. The input signals are speech sequences.

**Figure 15:**
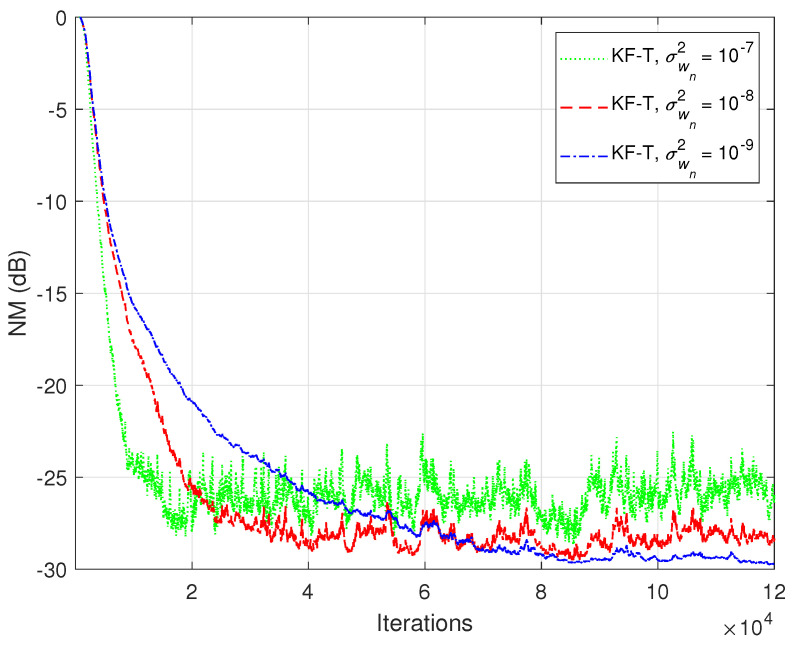
Normalized misalignment (NM) of the KF-T using different values of $\sigma_{w_{n}}^{2},\;n=1,2,\ldots ,N$, for the identification of the global impulse response, h(t)+u(t), where u(t) is randomly generated (Gaussian distribution), with variance $\upsilon {∥h(t)∥}^{2}/L$, using $\upsilon =10^{-3}$. The input signals are speech sequences.

The previous experiment is repeated in Figure 14, but using real-world speech sequences (corrupted by background noise) as input signals. Due to the nonstationary nature and highly correlated character of the speech signals, the performance of any adaptive algorithm is influenced in such a scenario. This is also the case for the KF-T, which pays with a slower convergence rate, as compared to the case analyzed in Figure 12. Nevertheless, this performance criterion can be improved by using higher values for the uncertainty parameters, $\sigma_{w_{n}}^{2},\;n=1,2,\ldots ,N$. This is further supported in Figure 15, where we can notice a higher convergence rate of the KF-T when increasing the values of $\sigma_{w_{n}}^{2}$, while causing a slight increase in the misalignment level.

## 6. Conclusions

In this paper, we have presented a tensorial Kalman filter tailored to the identification of multilinear forms. The solution was developed in the framework of a MISO system, while the identification problem was reformulated based on linearly separable systems modeled as rank-1 tensors. We have also shown how the resulting KF-T algorithm is connected to the main categories of tensor-based adaptive filters, i.e., the NLMS-T and the RLS-T algorithms. In this context, the specific uncertainty parameters of the KF-T allow for better control of this, as compared to its counterparts. The simulation results indicated the good performance features of the KF-T and also supported the discussion related to its connection with other tensorial algorithms. Future works will focus on finding a more practical way to evaluate the uncertainty parameters, e.g., following a similar approach to variable adaptation factors, which act as time-dependent parameters. In addition, we aim to extend the decomposition-based technique to the identification of nonseparable systems (which could be modeled as higher rank tensors), in order to develop a more general and efficient version of the tensorial Kalman filter.

## Figures and Tables

**Table 1:** Tensor-based Kalman filter (KF-T) for multilinear forms.

$$\begin{array}{c} \text{Inputs}:\\ x(t),d(t)\\ \text{Initialization}:\\ {\hat{h}}_{1}\left(0\right)={\left[\begin{array}{cccc} 1 & 0 & \cdots & 0 \end{array}\right]}^{T}\\ {\hat{h}}_{k}\left(0\right)=\frac{1}{L_{k}}{\left[\begin{array}{cccc} 1 & 1 & \cdots & 1 \end{array}\right]}^{T},\;k=2,\ldots ,N\\ R_{\mu_{h_{n}}}\left(0\right)=\varepsilon_{n}I_{L_{n}},\;\varepsilon_{n}>0,\;n=1,2,\ldots ,N\\ \text{Parameters}:\;\sigma_{v}^{2}\;\text{and}\;\sigma_{w_{n}}^{2},\;n=1,2,\ldots ,N\\ \text{For discrete-time index}\;t=1,2,\ldots \\ \;\;\;\;\;\text{For}\;n=1,2,\ldots ,N \end{array}$$
Equations (14)–(16)
$$\begin{array}{c} \;\;\;\;\;\;\;\;\;\;\;\;\;\;\;\;\;\;\;\;\;\;\;\;\;\;\;\;\;\;\;\;\;\;\;\;\;x_{{\hat{h}}_{1}{\hat{h}}_{2}\cdots {\hat{h}}_{n-1}{\hat{h}}_{n+1}\cdots {\hat{h}}_{N}}\left(t\right)=\left[{\hat{h}}_{N}\left(t-1\right)⊗{\hat{h}}_{N-1}\left(t-1\right)⊗\cdots ⊗{\hat{h}}_{n+1}\left(t-1\right)⊗I_{L_{n}}\right.\\ \;\;\;\;\;\;\;\;\;\;\;\;\;\;\;\;\;\;\;\;\;\;\;\;\;\;\;\;\;\;\;\;\;\;\;\;\;\;\;⊗{\hat{h}}_{n-1}\left(t-1\right)⊗{\left.\cdots ⊗{\hat{h}}_{2}\left(t-1\right)⊗{\hat{h}}_{1}\left(t-1\right)\right]}^{T}x\left(t\right) \end{array}$$
Equations (11)–(13)
$$\begin{array}{c} \;\;\;\;\;\hat{y}\left(t\right)={\hat{h}}_{n}^{T}\left(t-1\right)x_{{\hat{h}}_{1}{\hat{h}}_{2}\cdots {\hat{h}}_{n-1}{\hat{h}}_{n+1}\cdots {\hat{h}}_{N}}\left(t\right)\\ \;\;\;\;\;e\left(t\right)=d\left(t\right)-\hat{y}\left(t\right) \end{array}$$
Equation (20)
$$\begin{array}{c} R_{m_{h_{n}}}\left(t\right)=R_{\mu_{h_{n}}}\left(t-1\right)+\sigma_{w_{n}}^{2}I_{L_{n}} \end{array}$$
Equations (27)–(29)
$$\begin{array}{c} \;\;\;\;\;\;\;\;\;\;\;\;\;\;\;\;\;\;\;\;\;\;\;\;\;\;\;\;\;\;\;\;\;\;\;\;\;\;k_{{\hat{h}}_{n}}\left(t\right)=\frac{R_{m_{h_{n}}}\left(t\right)x_{{\hat{h}}_{1}{\hat{h}}_{2}\cdots {\hat{h}}_{n-1}{\hat{h}}_{n+1}\cdots {\hat{h}}_{N}}\left(t\right)}{x_{{\hat{h}}_{1}{\hat{h}}_{2}\cdots {\hat{h}}_{n-1}{\hat{h}}_{n+1}\cdots {\hat{h}}_{N}}^{T}\left(t\right)R_{m_{h_{n}}}\left(t\right)x_{{\hat{h}}_{1}{\hat{h}}_{2}\cdots {\hat{h}}_{n-1}{\hat{h}}_{n+1}\cdots {\hat{h}}_{N}}\left(t\right)+\sigma_{v}^{2}} \end{array}$$
Equations (30)–(32)
$$\begin{array}{c} \;\;\;\;\;\;\;\;\;\;\;\;\;\;\;\;\;\;\;\;\;\;\;R_{\mu_{h_{n}}}\left(t\right)=\left[I_{L_{n}}-k_{{\hat{h}}_{n}}\left(t\right)x_{{\hat{h}}_{1}{\hat{h}}_{2}\cdots {\hat{h}}_{n-1}{\hat{h}}_{n+1}\cdots {\hat{h}}_{N}}^{T}\left(t\right)\right]R_{m_{h_{n}}}\left(t\right) \end{array}$$
Equations (21)–(23)
$$\begin{array}{c} {\hat{h}}_{n}\left(t\right)={\hat{h}}_{n}\left(t-1\right)+k_{{\hat{h}}_{n}}\left(t\right)e\left(t\right) \end{array}$$
Equations (10)
$$\begin{array}{c} \hat{h}\left(t\right)={\hat{h}}_{N}\left(t\right)⊗{\hat{h}}_{N-1}\left(t\right)⊗\cdots ⊗{\hat{h}}_{2}\left(t\right)⊗{\hat{h}}_{1}\left(t\right)\\ \text{Outputs}:\\ \hat{y}\left(t\right),\;\hat{h}\left(t\right),\;{\hat{h}}_{n}\left(t\right),\;n=1,2,\ldots ,N. \end{array}$$

## Data Availability

The data presented in this study are available on request from the corresponding author.
